# Changes in Soil Chemical Attributes in an Agrosilvopastoral System Six Years After Thinning of Eucalyptus

**DOI:** 10.3390/plants13213050

**Published:** 2024-10-31

**Authors:** Wander Luis Barbosa Borges, Marcelo Andreotti, Luan Carlos Pianta da Cruz, Douglas Yuri Osaki de Oliveira, João Francisco Borges, Laryssa de Castro Silva

**Affiliations:** 1 Centro Avançado de Pesquisa e Desenvolvimento de Seringueira e Sistemas Agroflorestais, Instituto Agronômico, Votuporanga, São Paulo 15505-970, Brazil; 2 Faculdade de Engenharia, Universidade Estadual Paulista, Ilha Solteira, São Paulo 15385-000, Brazil; marcelo.andreotti@unesp.br; 3 Centro Universitário de Votuporanga, Votuporanga, São Paulo 15503-005, Brazil; luanpianta@yahoo.com.br (L.C.P.d.C.); dodoosaki191@gmail.com (D.Y.O.d.O.); jbfrancisco.borges@gmail.com (J.F.B.); laryssa75@hotmail.com (L.d.C.S.)

**Keywords:** fertility, intercropped systems, sustainable crop systems

## Abstract

The changes in soil chemical attributes in agrosilvopastoral systems after the thinning of trees are unclear. To address this gap, this study evaluated the effects of the thinning of eucalyptus hybrid Urograndis H-13 (*Eucalyptus urophylla* S. T. Blake × *E. grandis* W. Hill ex Maiden) on soil chemical fertility in an agrosilvopastoral system in an Arenic Hapludalf in Brazil. The experimental design was a randomized block with a 3 × 4 factorial design comprising three treatments (thinning of 0%, 50%, or 100% of the eucalyptus trees) and four sampling positions relative to the eucalyptus line (0, 2.0, 4.0, and 6.0 m). Six years after eucalyptus thinning, soil acidification was observed in the 0% and 50% eucalyptus thinning treatments, especially at 0 and 2 m from the eucalyptus line. Decreases in soil pH were associated with increases in the total acidity pH 7.0 (H^+^ + Al^3+^) and Al^3+^ content and decreases in the K^+^, Ca^2+^, and Mg^2+^ contents and base saturation over the soil profile (0–1.0 m).

## 1. Introduction

Integrated agricultural production systems are attractive options for addressing global issues such as food security, climate change, sustainable farming, and rural societal conditions [1]. The implementation of these systems is increasing in the Brazilian Cerrado biome [2], mainly in sandy soils. These systems include agrosilvopastoral systems, which comprise the simultaneous cultivation of grain-producing plants, trees, and forage in the same area to increase yields, economic stability, and crop diversity [3].

Integrating grain, forest, and animal production in the same area, especially under no-tillage, can preserve soil fertility through positive synergistic effects on the soil physical, chemical, and biological attributes, thereby reducing the soil degradation compared with single land-use systems, increasing crop yields, and reducing the environmental impact of production [3,4,5,6]. The tropical forages used in these systems have vigorous, deep root systems that increase nutrient cycling [7,8,9]. The addition of trees can also improve nutrient cycling, as the tree roots capture nutrients at greater depths that are not accessible to agricultural and forage crops [10].

One of the most common agrosilvopastoral systems in Brazil is the intercropping of soybean [*Glycine max* (L.) Merr.] or maize (*Zea mays* L.) with eucalyptus (*Eucalyptus* sp.) in the first and second years followed by the intercropping of tropical forages, such as palisade grass (*Urochloa brizantha* syn. *Brachiaria brizantha* cultivar Marandu), with eucalyptus. Unlike eucalyptus monoculture systems, these agrosilvopastoral systems require the pruning and thinning of the eucalyptus to promote the light input between the trees and reduce the competition for water and nutrients with soybean, maize, and tropical forages [11]. The animals in agrosilvopastoral systems tend to move near the tree planting line, which can affect soil chemical attributes in these areas due to higher trampling and the deposition of feces and urine [1]. In addition, the inclusion of species with different root systems and plant residues with different C/N ratios can alter the decomposition and nutrient cycling rates [12]. However, the nature of these changes, especially after the thinning of eucalyptus, is unclear. To address this gap in knowledge, in this study, we tested the following hypotheses: (a) the effects of the thinning of eucalyptus in agrosilvopastoral systems on soil chemical attributes throughout the soil profile differ depending on the percentage of thinning, and (b) the effects of hte thinning of eucalyptus in agrosilvopastoral systems on soil chemical attributes throughout the soil profile differ depending on the distance from the tree planting line.

## 2. Results

On 14 April 2015 (approximately one year before eucalyptus thinning), the palisade grass dry matter yield was 1.825 and 2.870 kg ha^−1^ higher at 4.0 and 6.0 m from the eucalyptus line than at 0 m, respectively [13]. After eucalyptus thinning, the soybean grain yield was 980–1396 kg ha^−1^ higher in the 100% eucalyptus thinning treatment than in the 0% eucalyptus thinning treatment [11]. The maize grain yield was 7.088–8.401 kg ha^−1^ higher in the 100% eucalyptus thinning treatment than in the 0% eucalyptus thinning treatment [11].

**Figure 1:**
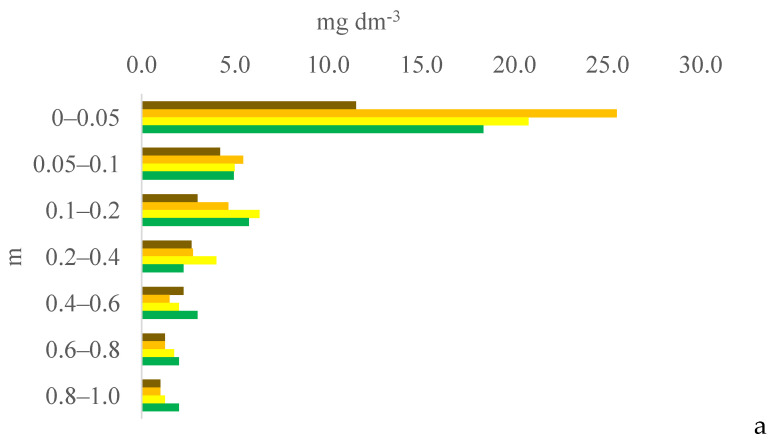

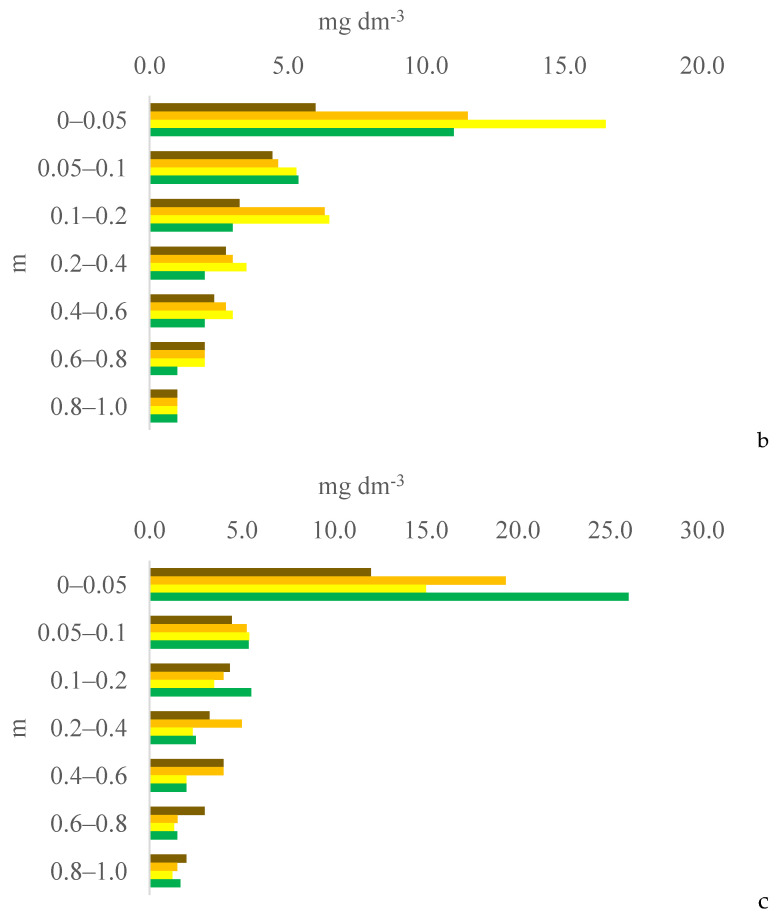
Interaction between eucalyptus thinning of 0% (**a**), 50% (**b**), or 100% (**c**) and sampling position relative to the eucalyptus line: 0 m (brown); 2.0 m (orange); 4.0 m (yellow); and 6.0 m (green) for soil P content at depths of 0–0.05, 0.05–0.1, 0.1–0.2, 0.2–0.4, 0.4–0.6, 0.6–0.8, and 0.8–1.0 m.

**Figure 2:**
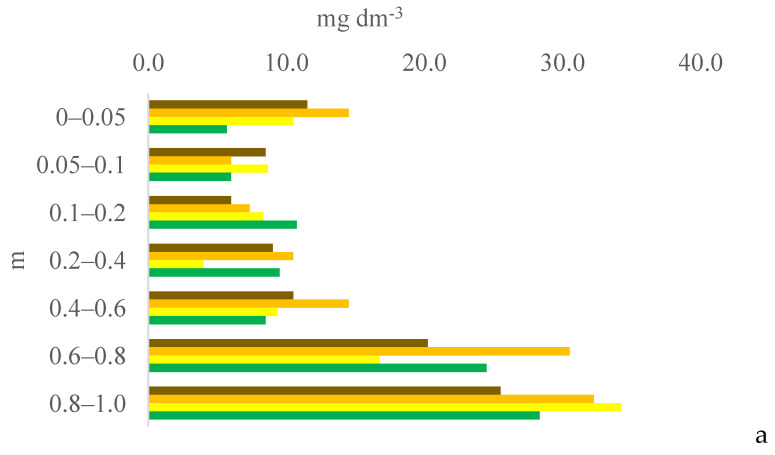

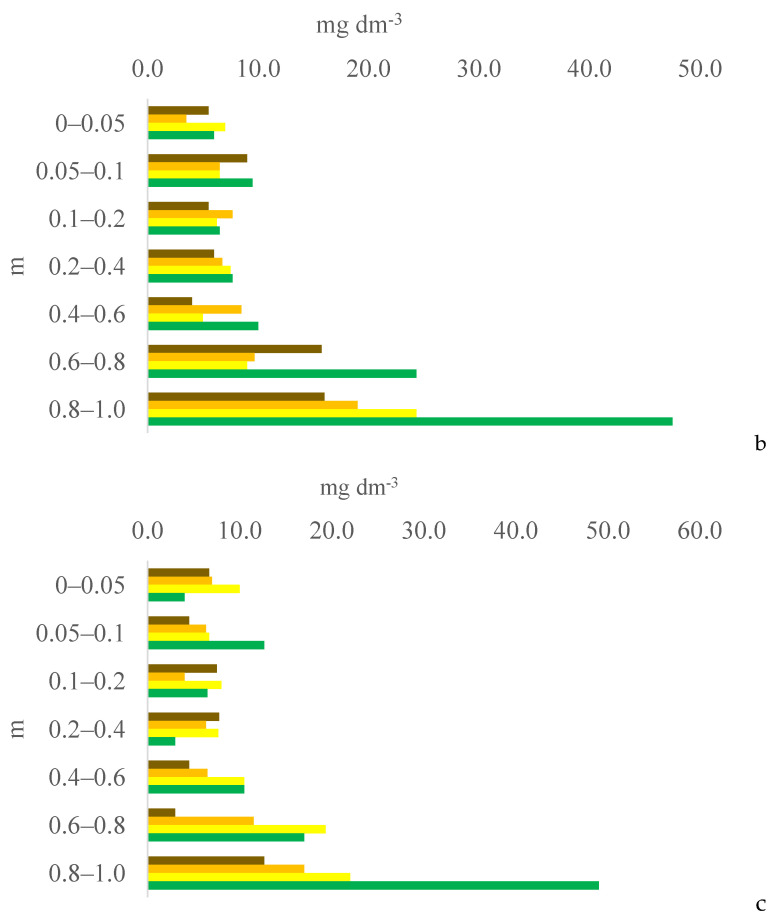
Interaction between eucalyptus thinning of 0% (**a**), 50% (**b**), or 100% (**c**) and sampling position relative to the eucalyptus line: 0 m (brown); 2.0 m (orange); 4.0 m (yellow); and 6.0 m (green) for soil S content at depths of 0–0.05, 0.05–0.1, 0.1–0.2, 0.2–0.4, 0.4–0.6, 0.6–0.8, and 0.8–1.0 m.

**Figure 3:**
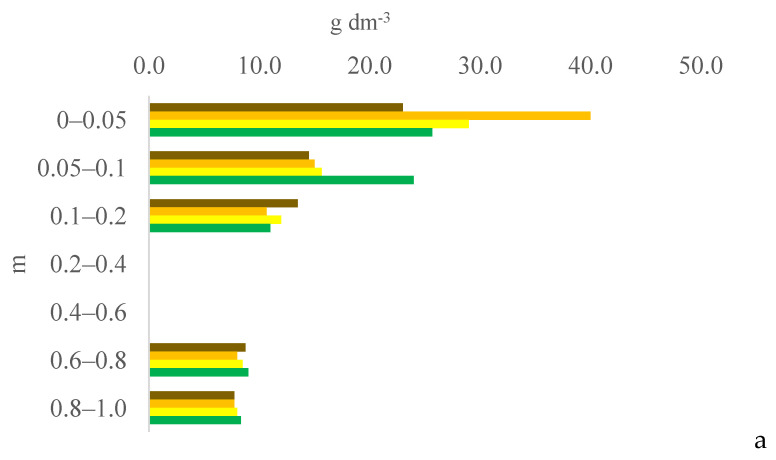

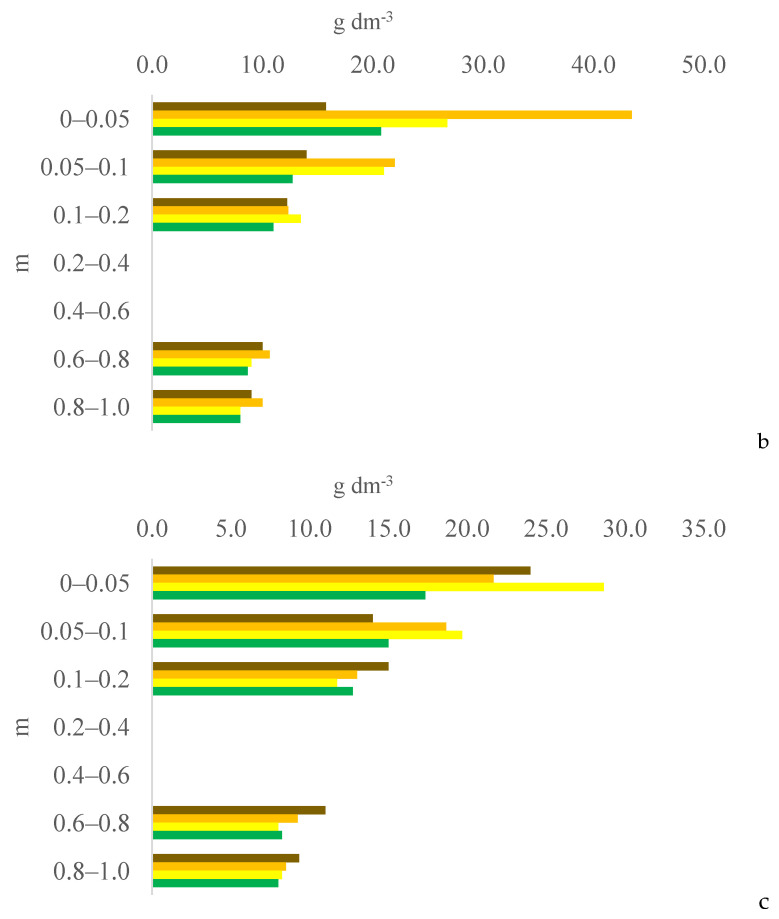
Interaction between eucalyptus thinning of 0% (**a**), 50% (**b**), or 100% (**c**) and sampling position relative to the eucalyptus line: 0 m (brown); 2.0 m (orange); 4.0 m (yellow); and 6.0 m (green) for soil OM content at depths of 0–0.05, 0.05–0.1, 0.1–0.2, 0.2–0.4, 0.4–0.6, 0.6–0.8, and 0.8–1.0 m.

**Figure 4:**
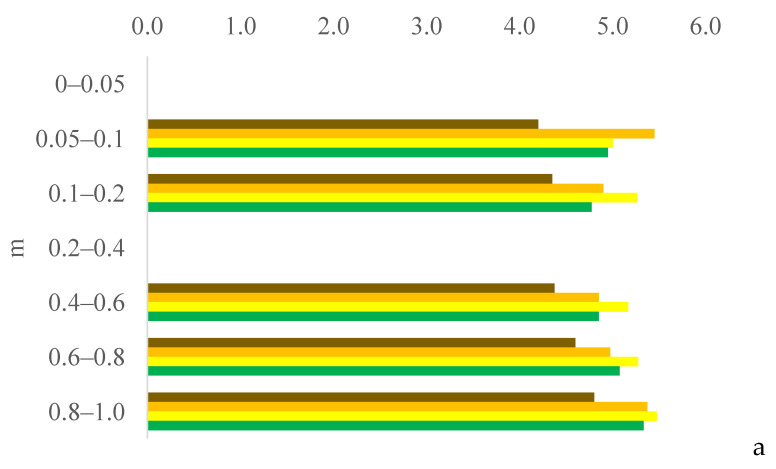

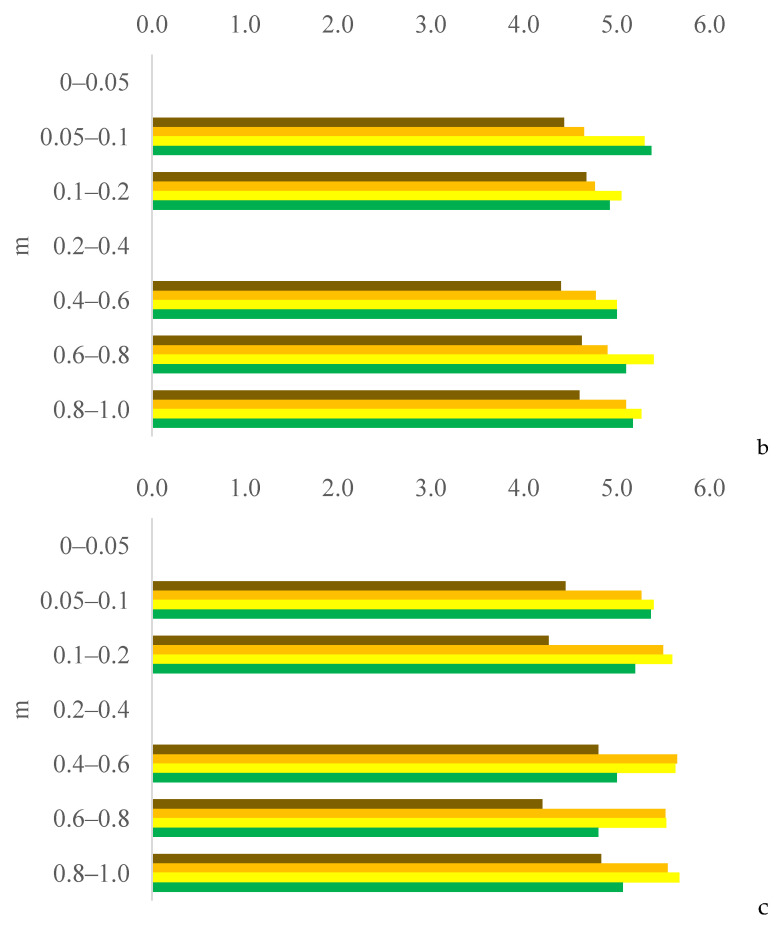
Interaction between eucalyptus thinning of 0% (**a**), 50% (**b**), or 100% (**c**) and sampling position relative to the eucalyptus line: 0 m (brown); 2.0 m (orange); 4.0 m (yellow); and 6.0 m (green) for soil pH at depths of 0–0.05, 0.05–0.1, 0.1–0.2, 0.2–0.4, 0.4–0.6, 0.6–0.8, and 0.8–1.0 m.

**Figure 5:**
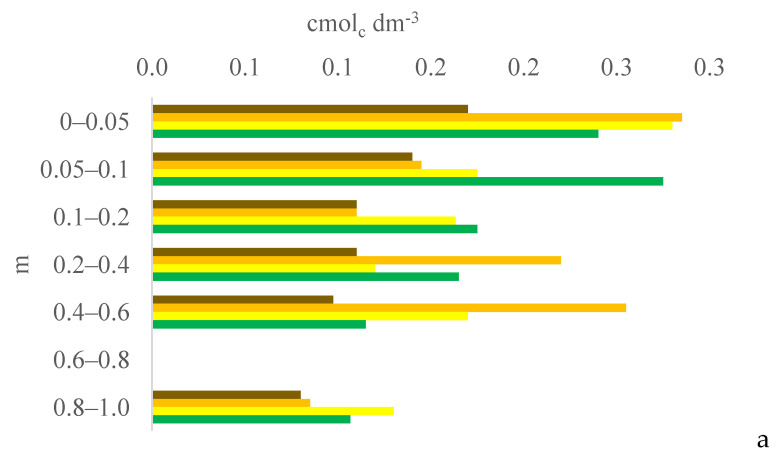

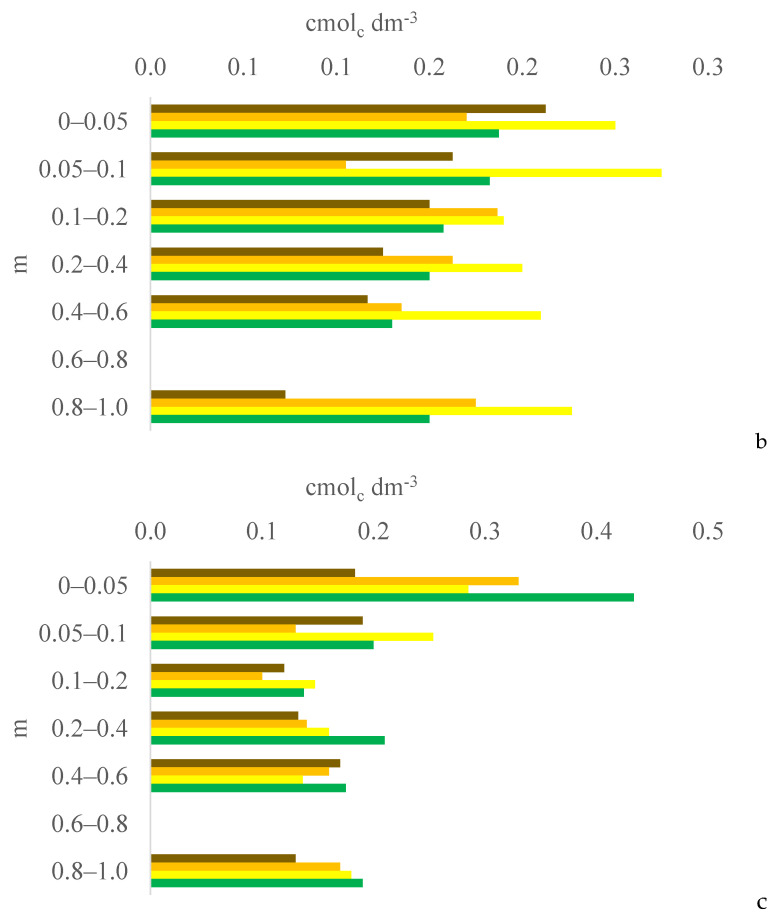
Interaction between eucalyptus thinning of 0% (**a**), 50% (**b**), or 100% (**c**) and sampling position relative to the eucalyptus line: 0 m (brown); 2.0 m (orange); 4.0 m (yellow); and 6.0 m (green) for soil K^+^ content at depths of 0–0.05, 0.05–0.1, 0.1–0.2, 0.2–0.4, 0.4–0.6, 0.6–0.8, and 0.8–1.0 m.

**Figure 6:**
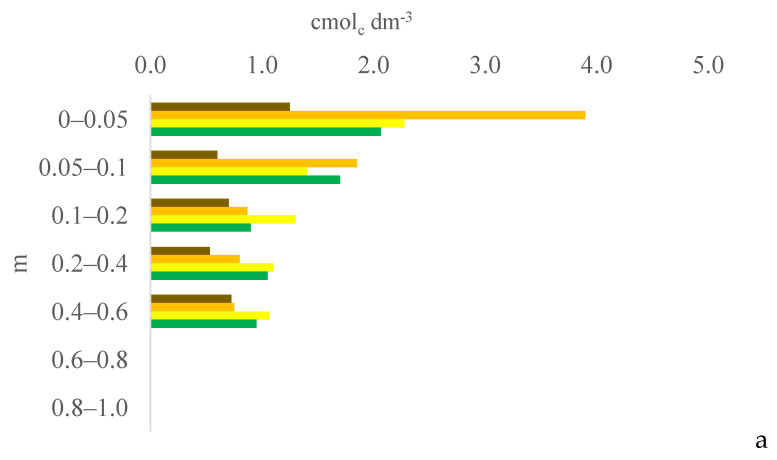

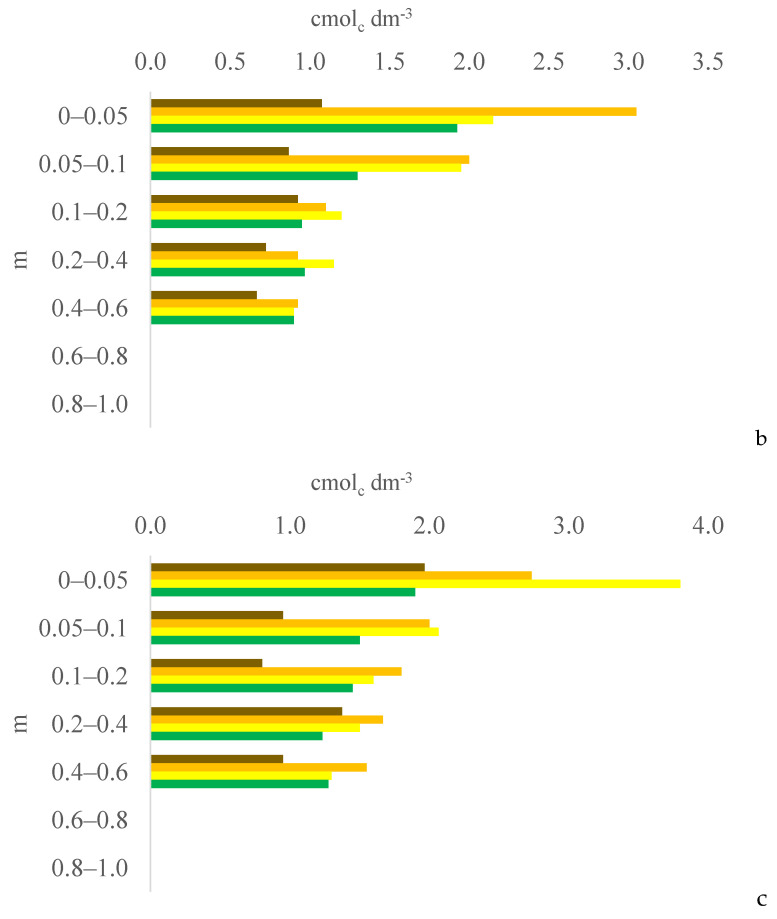
Interaction between eucalyptus thinning of 0% (**a**), 50% (**b**), or 100% (**c**) and sampling position relative to the eucalyptus line: 0 m (brown); 2.0 m (orange); 4.0 m (yellow); and 6.0 m (green) for soil Ca^2+^ content at depths of 0–0.05, 0.05–0.1, 0.1–0.2, 0.2–0.4, 0.4–0.6, 0.6–0.8, and 0.8–1.0 m.

**Figure 7:**
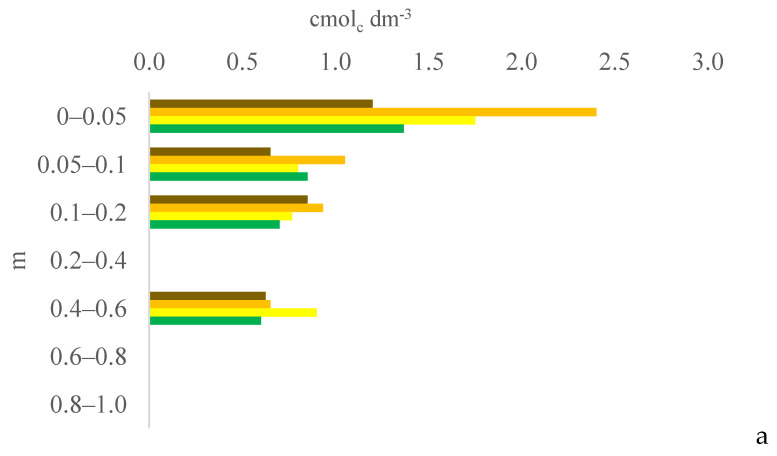

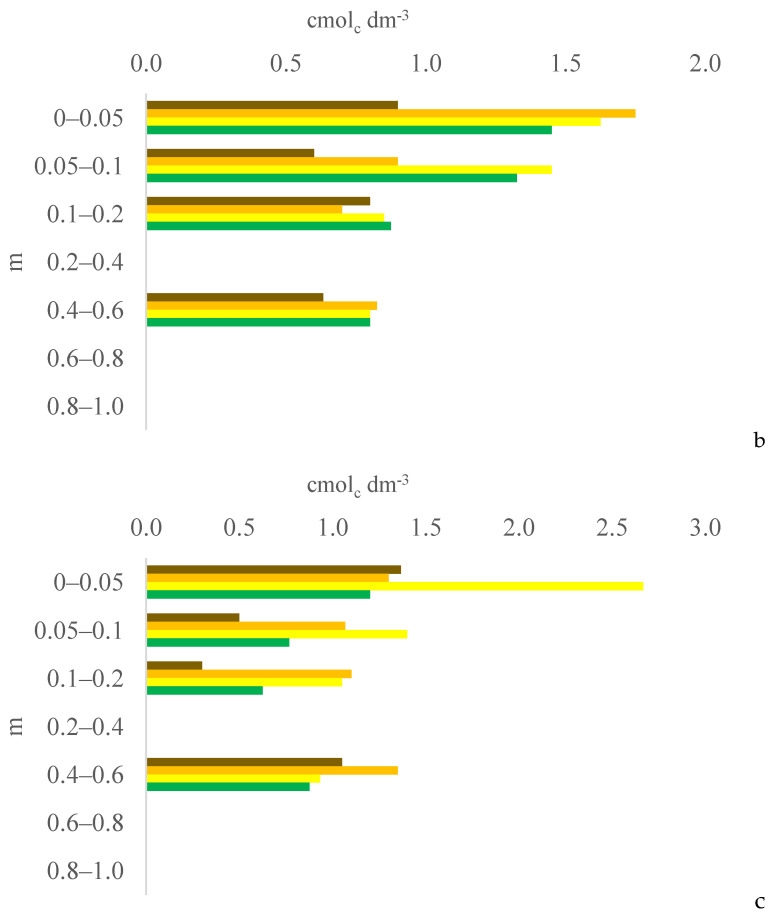
Interaction between eucalyptus thinning of 0% (**a**), 50% (**b**), or 100% (**c**) and sampling position relative to the eucalyptus line: 0 m (brown); 2.0 m (orange); 4.0 m (yellow); and 6.0 m (green) for soil Mg^2+^ content at depths of 0–0.05, 0.05–0.1, 0.1–0.2, 0.2–0.4, 0.4–0.6, 0.6–0.8, and 0.8–1.0 m.

**Figure 8:**
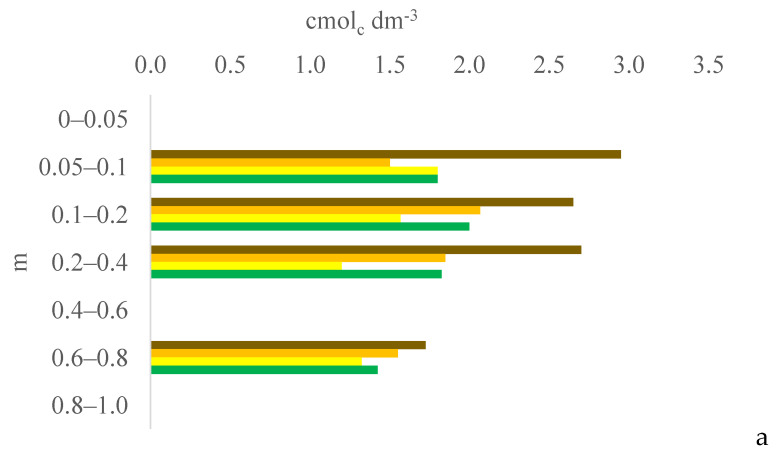

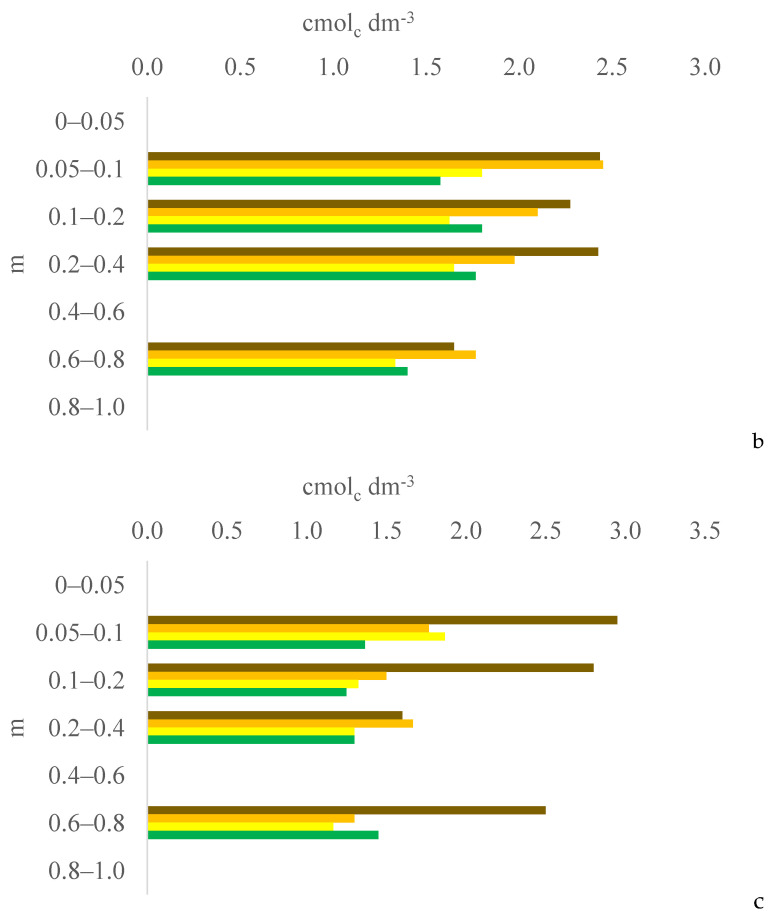
Interaction between eucalyptus thinning of 0% (**a**), 50% (**b**), or 100% (**c**) and sampling position relative to the eucalyptus line: 0 m (brown); 2.0 m (orange); 4.0 m (yellow); and 6.0 m (green) for soil total acidity pH 7.0 (H^+^ + Al^3+^) at depths of 0–0.05, 0.05–0.1, 0.1–0.2, 0.2–0.4, 0.4–0.6, 0.6–0.8, and 0.8–1.0 m.

**Figure 9:**
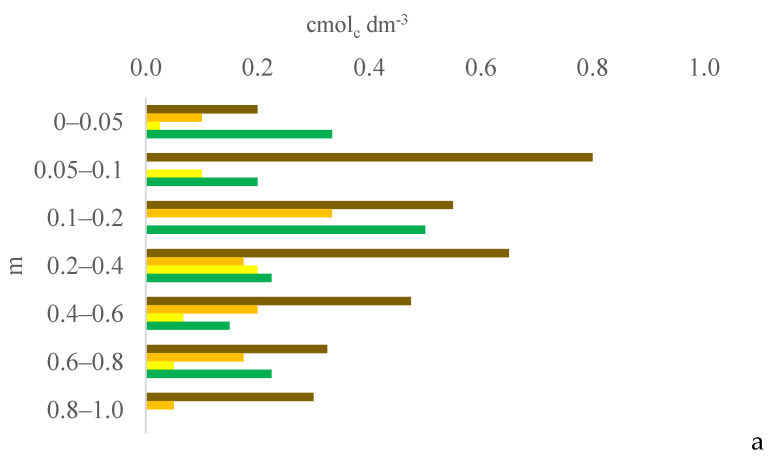

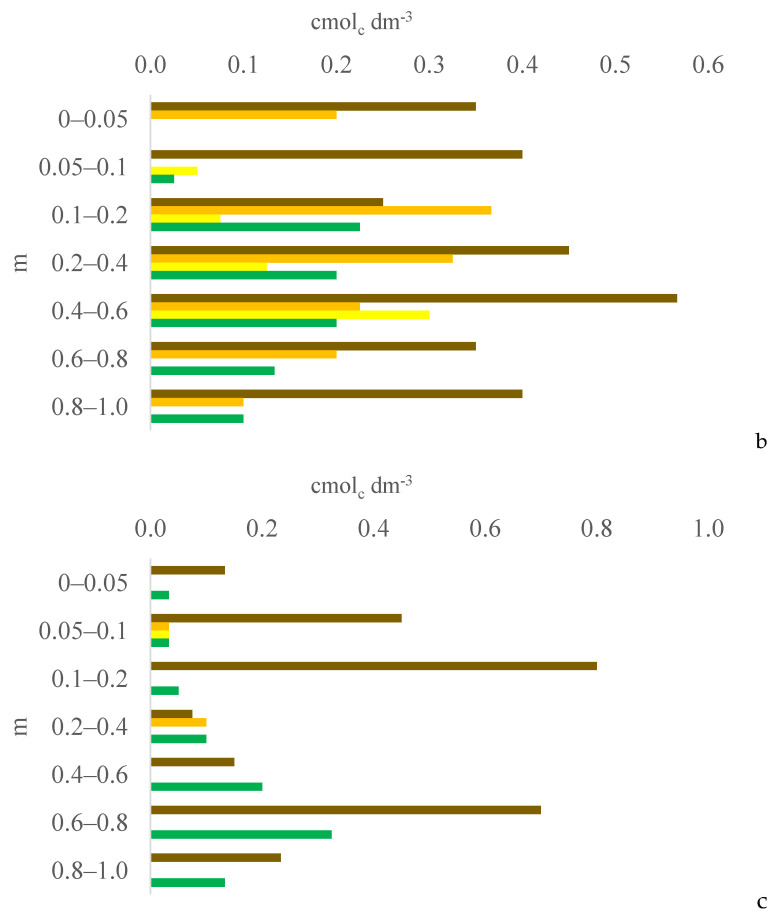
Interaction between eucalyptus thinning of 0% (**a**), 50% (**b**), or 100% (**c**) and sampling position relative to the eucalyptus line: 0 m (brown); 2.0 m (orange); 4.0 m (yellow); and 6.0 m (green) for soil Al^3+^ content at depths of 0–0.05, 0.05–0.1, 0.1–0.2, 0.2–0.4, 0.4–0.6, 0.6–0.8, and 0.8–1.0 m.

**Figure 10:**
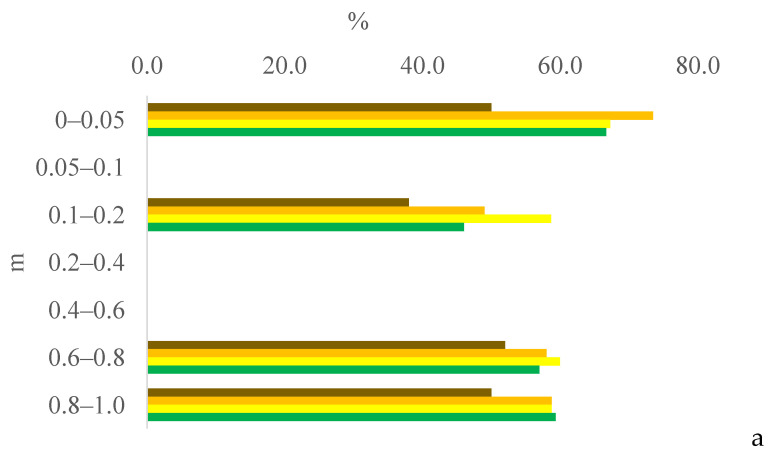

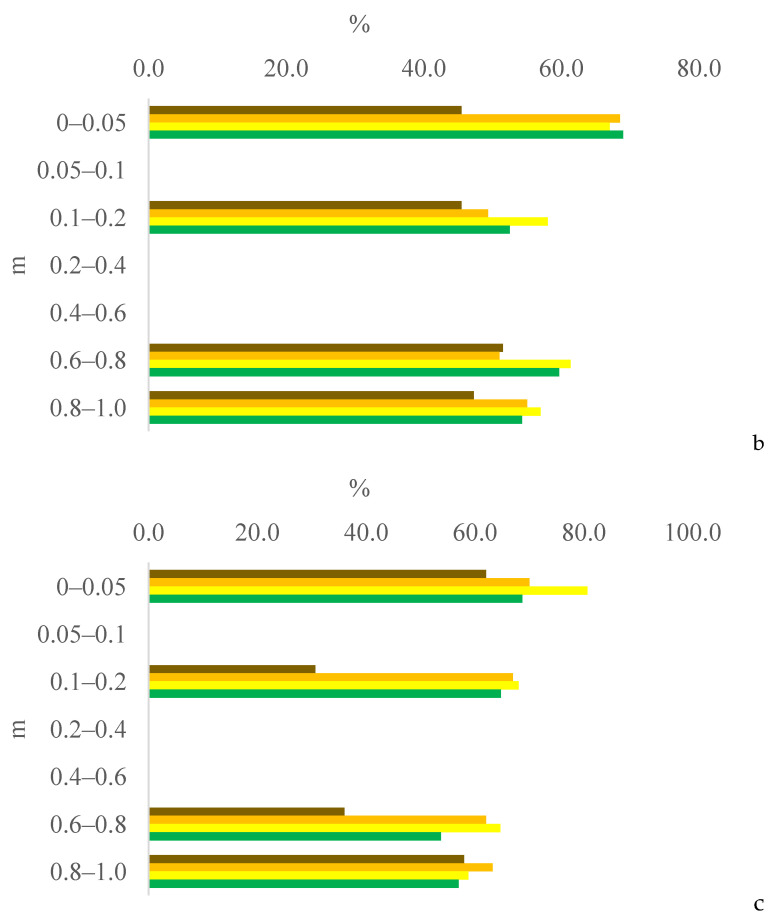
Interaction between eucalyptus thinning of 0% (**a**), 50% (**b**), or 100% (**c**) and sampling position relative to the eucalyptus line: 0 m (brown); 2.0 m (orange); 4.0 m (yellow); and 6.0 m (green) for soil BS at depths of 0–0.05, 0.05–0.1, 0.1–0.2, 0.2–0.4, 0.4–0.6, 0.6–0.8, and 0.8–1.0 m.

Six years after eucalyptus thinning, the changes in soil chemical attributes were greatest in the 100% eucalyptus thinning treatment. The values of the F test for soil chemical attributes are provided in Table S1 for the 0–0.05, 0.05–0.1, and 0.1–0.2 m layers; Table S2 for the 0.2–0.4, 0.4–0.6, and 0.6–0.8 m layers; and Table S3 for the 0.8–1.0 m layer, in the Supplementary Files. Figure 1, Figure 2, Figure 3, Figure 4, Figure 5, Figure 6, Figure 7, Figure 8, Figure 9 and Figure 10 illustrate the interactions between the percentage of trees thinned and sampling position relative to the eucalyptus line for P (Figure 1); S (Figure 2); organic matter (OM) (Figure 3); pH (Figure 4); K^+^ (Figure 5); Ca^2+^ (Figure 6); Mg^2+^ (Figure 7); H^+^ + Al^3+^ (Figure 8); Al^3+^ (Figure 9); and BS (Figure 10) at depths of 0–0.05, 0.05–0.1, 0.1–0.2, 0.2–0.4, 0.4–0.6, 0.6–0.8, and 0.8–1.0 m. The soil chemical attributes throughout the soil profile were significantly affected (P < 0.05) by the distance from the eucalyptus planting line and percentages of thinning.

### 2.1. 0% Eucalyptus Thinning Treatment

Compared to the 50% and 100% eucalyptus thinning treatments, standard tree maintenance (thinning of 0% of the eucalyptus trees) increased the S content in the 0–0.05 and 0.2–1.0 m layers at 2.0 m from the eucalyptus line (2.0 m). By contrast, at 6.0 m from the eucalyptus line (6.0 m), 0% thinning decreased the soil pH compared to 50% and 100% thinning, leading to a decrease in the K^+^ content in the 0.05–0.1 m layer and an increase in the Al^3+^ content in the 0–0.2 m layer.

Regarding the sampling positions relative to the eucalyptus line in the 0% thinning treatment, the soil pH was lower at 0 m from the eucalyptus line (0 m) than at 2.0 m, 4.0 m, and 6.0. The decrease in soil pH was associated with increases in the Al^3+^ content in the 0.05–0.1, 0.4–0.6, and 0.8–1.0 m layers; a decrease in the Ca^2+^ content and an increase in the soil total acidity pH 7.0 (H^+^ + Al^3+^) in the 0.05–0.1 m layer; and decreases in the BS in the 0–0.05 and 0.8–1.0 m layers.

### 2.2. 50% Eucalyptus Thinning Treatment

Compared to the 0% and 100% eucalyptus thinning treatments, the removal of 50% of the trees (50% thinning) reduced the P content in the 0–0.1 m layer at 2 m and in the 0–0.05 and 0.1–0.2 m layers at 6 m; reduced the S content in the 0.4–0.8 m layer at 4 m; decreased the soil pH and increased the total acidity in the 0.05–0.1 m layer at 2 m; and increased the Al^3+^ content in the 0–0.05, 0.4–0.6, and 0.8–1.0 m layers at 0 m.

The Soil pH in the 50% thinning treatment was lower at 0 m than at 2.0, 4.0, and 6.0 m, leading to an increased Al^3+^ content in the 0–0.1, 0.4–0.6, 0.6–0.8 m, and 0.8–1.0 m layers; a decreased K^+^ content in the 0.8–1.0 m layer; and a lower BS in the 0–0.05 m and 0.8–1.0 m layers.

### 2.3. 100% Eucalyptus Thinning Treatment

Compared to the 0% and 50% eucalyptus thinning treatments, the removal of 100% of the trees (100% thinning) increased the P content in the 0.4–1.0 m layer at 0 m, the 0.2–0.6 m layer at 2 m, and the 0–0.05 layer at 6 m, and increased the S content in the 0.05–0.1 layer at 6 m. At all distances from the eucalyptus line (0, 2.0, 4.0, and 6.0 m), 100% thinning increased the soil pH and decreased the total acidity in the 0–0.05 m layer; in addition, 100% thinning increased the soil pH and increased the Mg^2+^ content in the 0.2–0.4 m layer. At 0 m, 100% thinning decreased the Al^3+^ content and increased the BS in the 0–0.05 m layer; decreased the total acidity and Al^3+^ content and increased the BS in the 0.2–0.4 m layer; increased the soil pH, K^+^ and Mg^2+^ contents, and the BS and decreased the Al^3+^ content in the 0.4–0.6 m layer; and increased the K^+^ content and BS in the 0.8–1.0 m layer. At 2 m, 100% thinning increased the soil pH and Ca^2+^ content and decreased the Al^3+^ content in the 0.1–0.2 layer; increased the BS in the 0.1–0.2 m layer; increased the Ca^2+^ content in the 0.2–0.4 m layer; increased the soil pH and Ca^2+^ and Mg^2+^ contents and decreased the Al^3+^ content in the 0.4–0.6 m layer; increased the pH and decreased the Al^3+^ content in the 0.6–0.8 m layer; and decreased the Al^3+^ content in the 0.8–1.0 m layer. At 4 m, 100% thinning increased the Ca^2+^ and Mg^2+^ contents and BS in the 0–0.05 m layer and increased the soil pH in the 0.4–0.6 m layer. At 6 m, 100% thinning increased the K^+^ content in the 0–0.05 layer; increased the Ca^2+^ content and decreased the Al^3+^ content in the 0.1–0.2 m layer; and increased the K^+^ and Ca^2+^ contents in the 0.4–0.6 m layer. Overall, as the pH increased, the P, S, Ca^2+^, Mg^2+^, and K^+^ contents increased, while the H^+^ + Al^3+^ and Al contents decreased. In addition, increases in the Ca^2+^, Mg^2+^, and K^+^ contents were associated with increases in the BS, whereas increases in the H^+^ + Al^3+^ and Al^3+^ contents led to decreases in the BS.

With respect to the sampling positions relative to the eucalyptus line in the 100% thinning treatment, the soil pH and Ca^2+^ content increased and the total acidity and Al^3+^ content decreased in the 0.05–0.2 m layer at 0 m compared to 2.0, 4.0, and 6.0 m. In addition, at 0 m, the K^+^ content decreased and the Al^3+^ content increased in the 0–0.05 m layer; the P content decreased in the 0.05–0.1 m layer; the Mg^2+^ content and BS decreased in the 0.1–0.2 m layer; the Ca^2+^ content decreased in the 0.4–0.6 m layer; the soil pH, BS, and S content decreased and the total acidity and Al^3+^ content increased in the 0.6–0.8 m layer; and the Al^3+^ content increased in the 0.8–1.0 m layer. By contrast, at 6.0 m, the K^+^ and S contents increased in the 0–0.05 m layer, and the S content increased in the 0.8–1.0 m layer.

## 3. Discussion

The changes in soil chemical attributes in the surface and subsurface layers differed depending on the percentage of eucalyptus thinning and were most pronounced near the tree planting line (0 and 2 m), confirming our hypotheses. These differences reflected the effects of the decomposition of the residues from the aerial parts and roots of soybean, maize, and palisade grass and the decomposition of eucalyptus litter and roots in the agrosilvopastoral system.

### 3.1. Soil P Content

Among the sampled soil depths, the soil P content was highest in the surface layer (0–0.05 m) regardless of the percentage of thinning or the distance from the eucalyptus line. The soil in the experimental area has not been tilled since the 2009–10 season, promoting nutrient accumulation in the surface layers [14].

At 0 m from the eucalyptus line, the soil P content in the subsurface layers (0.4–0.6 and 0.8–1.0 m) was higher in the 100% treatment than in the 0% and 50% treatments. However, in contrast to our expectations, the soil P content at 2, 4, or 6 m from the eucalyptus line was not higher in the 100% treatment than in the 0% and 50% treatments. In the 0% and 50% treatments, the constant supply of organic material by eucalyptus roots likely increased the organic P in deeper layers [15] and, consequently, increased the P content in the soil profile after mineralization.

### 3.2. Soil Organic Matter and S Contents

In the 100% treatment, the soil OM content in the 0.1–1.0 m layer was, on average, higher at 0 m from the eucalyptus line than at the other sampling distances due to the decomposition of eucalyptus litter accumulated as fallen leaves, branches, and twigs on the soil (plant material). This litter is characterized by a high C/N ratio and high levels of lignin and polyphenols, resulting in slow decomposition [16] and favoring the maintenance of OM in the soil [17]. The eucalyptus root system also contributed to the higher OM content at 0 m because no other species grew at this position in the transect [1]. This result is consistent with those of [17], who found that the eucalyptus root system increased the soil content of light OM, that is, recently deposited OM that has been altered slightly by humification. In addition, in agrosilvopastoral systems in tropical regions, animals cluster under the tree canopy in search of milder air temperatures, especially during the hottest months of the year [18,19]. This clustering increases the deposition of feces and urine (excrements) by grazing animals closer to the planting line (0 m). These excrements are considered organic fertilizers and gradually increase the soil OM content [20].

Mineralization gradually makes S available to the soil solution in the form of sulfate. Tropical soils are poor not only in the OM and P contents but also in the S content, and crop systems that increase the S content are of interest because S is essential for forage production. However, no clear trends were observed in the effects of the thinning percentage and distance from the eucalyptus line on the soil S content. In the 0% treatment, the soil S content was highest in the 0–0.05 and 0.4–0.6 m layers at 0 m and in the 0–0.05 and 0.2–1.0 m layers at 2.0 m due to the mineralization of OM accumulated at this site from plant material.

### 3.3. Soil pH and Total Acidity pH 7.0 (H^+^ + Al^3+^)

Although the accumulation of plant material on the soil and the higher concentration of eucalyptus roots at 0 m increased the soil OM and S contents, the decomposition of this material acidified the soil, decreasing the pH and increasing the total acidity. On average, the soil pH was lower and total acidity was higher in the surface and subsurface layers at 0 m than at 2.0, 4.0, and 6.0 m. According to [21], the decomposition of organic residues (plants) increases the acidity, and the mineralization of OM by soil microorganisms releases nitrate and H, further reducing the pH [22].

Cerrado biome soils like those in this experiment are naturally acidic. Soil acidity decreases the Ca^2+^, Mg^2+^, and K^+^ contents and increases the Al^3+^ content, which is toxic for plants. Consequently, crop systems that promote the soil buffering power can limit variations in soil pH. In this study, acidification in the surface and subsurface layers (0–0.05 and 0.2–0.4 m at 0, 2.0, 4.0, and 6.0 m and 0.1–0.2 and 0.4–0.8 m at 2.0 m) was lower in the 100% treatment than in the 0% and 50% treatments because the higher solar radiation in the 100% treatment increased the soybean and maize residue inputs [11,23] and, consequently, the buffering power of the soil. Solar radiation was lower in the 0% and 50% treatments due to greater shading; the intensity of shade in agrosilvopastoral systems decreases with the distance from the eucalyptus line [24]. Shade significantly influences the photosynthetic processes of plants, especially those of C4 cycle plants such as maize [25] and palisade grass. The reduction in photosynthetically active radiation can decrease the grain productivity of maize plants that are closer to trees [26]. Shade also reduces the air temperature and CO_2_ concentrations, further decreasing crop yields compared to crops that are completely exposed to the sun [27]. In addition, competition between eucalyptus and crops for nutrients and water can affect crop yields depending on the distance between the rows of trees [28]. Thus, incident solar radiation under the canopy is a strong determinant of the success of agricultural and/or forage production in agrosilvopastoral systems [29].

### 3.4. Soil K^+^, Ca^2+^, and Mg^2+^ Contents

The soil pH was lowest in the 0–1.0 m layer at 0 m in the 0% treatment (range of 4.2–4.83), which reduced the availability of K^+^, Ca^2+^, and Mg^2+^. The availability of these cations in soil decreases when the soil pH is less than 5.4 [30]. The low soil contents of K^+^ and Ca^2+^ in the soil profile (0–1.0 m layer) at 0 m from the planting line in the 0% treatment also reflect the greater presence of fine eucalyptus roots [8,31,32], which efficiently absorb nutrients during plant growth [1]. Plants absorb Ca^2+^ almost exclusively via the roots [33]. Ca^2+^ plays roles in root growth [34], including in cell division [35]. This may explain the higher soil Ca^2+^ content at 0 m in the 100% treatment than in the 0% treatment.

Compared with the 0% treatment, the 100% treatment increased the soil K+ content in the 0–0.05, 0.2–0.4, and 0.8–1.0 m layers; the Ca^2+^ content in the 0.1–0.6 and 0.8–1.0 m layers; and the Mg^2+^ content in the 0.2–0.6 m layers at 2.0, 4.0, and 6.0 m from the eucalyptus line. These increases were due to nutrient cycling by soybean and maize, which had greater grain yields under these conditions [11], and by palisade grass, which had a greater dry matter yield at 4.0 and 6.0 m [13]. Palisade grass has a voluminous fasciculate root system, and the regrowth of tillers after grazing increases the volume of soil pores, allowing the movement of K^+^, Ca^2+^, and Mg^2+^ throughout the soil profile [36,37]. Moreover, cation mobility in the soil profile is promoted by the formation of complexes between these cations and soluble organic compounds released by the decomposition of plant biomass deposited on the soil surface (carboxylic and phenolic radicals) and by the release of low-molecular-weight organic acids from root exudates, under the influence of grazing, and from the decomposition of animal waste, mainly feces [38,39,40]. In addition, the downward movement of Ca^2+^ through the root zone occurs via root canals [41].

### 3.5. Soil Al^3+^ Content

The lower pH at 0 m increased the Al^3+^ content at this distance from the eucalyptus line compared with distances of 2.0, 4.0, and 6.0 m. These increases occurred in both the surface and subsurface layers in the 0%, 50%, and 100% treatments (0–0.2 and 0.6–1.0 m in 0%; 0.05–0.1, 0.2–0.6, and 0.8–1.0 m in 50%; and 0–0.1 and 0.6–1.0 m in 100%). According to [42], the concentration of Al^3+^ in the soil solution increases with soil acidity. At a low pH, H^+^ acts on minerals to release Al^3+^, which is predominantly retained by negatively charged clay particles in the soil in equilibrium with Al^3+^ in the soil solution [22]. By contrast, in soils with pH >5.5, Al is found in precipitated forms [42,43].

### 3.6. Soil BS

The BS in the surface and subsurface layers was lower at 0 m than at 2.0, 4.0, and 6.0 m in all three eucalyptus thinning treatments (0.1–0.2 and 0.6–1.0 m in 0% and 0–0.05 and 0.8–1.0 m in the 50% and 100% treatments). These decreases were the result of lower soil contents of K^+^, Ca^2+^, and Mg^2+^ and a higher H^+^ + Al^3+^ content. Compared with the BS in the 0% and 50% treatments, the BS in the 100% treatment was higher in the 0.2–0.6 m layer at four positions (0, 2, 4, and 6 m) and in the 0–0.05 and 0.8–1.0 m layers at 0 m from the eucalyptus line. These increases were due to the higher soil Ca^2+^ and Mg^2+^ contents.

### 3.7. Considerations

The complete removal of eucalyptus trees (100% thinning) resulted in the highest soybean and maize grain yields and palisade grass dry matter yield and increased nutrient cycling. However, the height and diameter of the trees in the 0% and 50% thinning treatments increased in the six years after thinning, which is important for financial returns. Maintaining all trees (0% eucalyptus thinning treatment) or removing only 50% of the trees (50% eucalyptus thinning treatment) resulted in soil acidification, with increases in the soil pH, total acidity, and Al^3+^ content and decreases in the K^+^, Ca^2+^, and Mg^2+^ contents and BS throughout the soil profile (0–1.0 m), especially at 0 and 2 m from the eucalyptus line. These changes occurred due to the decomposition of eucalyptus litter and roots and the consumption of nutrients by the eucalyptus. Therefore, at 0% and 50% thinning, the soil fertility should be monitored to correct the surface and subsurface acidity and prevent damage to plant development.

## 4. Materials and Methods

### 4.1. Description of Site, Soil, Climate, and Treatments

The experiment began in May 2009 at the Advanced Research and Development Center for Rubber Tree and Agroforestry Systems of the Agronomic Institute (IAC) of the São Paulo Agency for Agribusiness Technology (APTA), which is located in the Cerrado biome in the municipality of Votuporanga, São Paulo State, Brazil (20°20′ S, 49°58′ W and 510 m altitude). The soil is Arenic Hapludult [44], hereinafter referred to as Ultisol, with a sandy texture. The site is a degraded area with a slope < 5% that was previously used as pasture for 10 consecutive years; the soil had been managed under conventional tillage with plowing and harrowing. The climate in the region is tropical with dry winters (Aw type according to Köppen’s classification). The average annual maximum, minimum, and mean temperatures are 31.2 °C, 17.4 °C, and 24 °C, respectively, and the average annual rainfall is 1328.6 mm. Soil samples were taken at depths of 0–0.2 and 0.2–0.4 m for chemical [45], physical [46], granulometric [47], and structural characterization [48], and the results are shown in Table 1.

The experimental design was randomized block with a 3 (percentages of thinning) × 4 (sampling positions) factorial design and four replications. The three treatments were different percentages of thinning of the eucalyptus hybrid Urograndis H-13: thinning of 0% of the trees (0%); thinning of 50% of the trees (50%); and thinning of 100% of the trees (100%). The four sampling positions were 0 m from the eucalyptus line (0 m); 2.0 m from the eucalyptus line (2.0 m); 4.0 m from the eucalyptus line (4.0 m); and 6.0 m from the eucalyptus line (6.0 m).

### 4.2. Crop Management

#### 4.2.1. 2009–10 Season

After tillage in September 2009, millet (*Pennisetum glaucum*) was sown between the terraces for soil conservation. In October 2009, the eucalyptus hybrids were planted on the terraces in a simple line system with a spacing of 2 m between trees (plants) and 13.5 m between rows and a density of 370 plants ha^−1^. On 30 November 2009, the millet was desiccated, and soybean was sown between the terraces over the millet straw under no-tillage. The soybean was harvested on April 8, 2010, and sunn hemp (*Crotalaria juncea*) was sown as a cover crop.

#### 4.2.2. 2010–11 Season

The sunn hemp was desiccated on 29 November 2010, and maize was sown between the terraces on the sunn hemp straw on 15 December 2010, under no-tillage. Palisade grass was sown on 16 December 2010; two rows were sown between rows of the maize crop. In September 2011, newly weaned beef cattle were introduced in the area and remained grazing in the area until slaughter. After the slaughter of the first group of cattle, new groups of beef cattle were introduced in the area and remained grazing in the area until slaughter. The stocking rate of the cattle varied according to the forage supply.

#### 4.2.3. 2015–16 Season

On 22 July 2016, the cattle were removed from the area for pasture regeneration. Thinning of the eucalyptus was carried out on 25–26 July 2016, by removing 0% of the trees, 50% of the trees, or 100% of the trees, depending on the treatment. At this time, the eucalyptus trees had an average height of 26.5 m and an average diameter at breast height of 0.26 m.

#### 4.2.4. 2016–17 Season

The area was desiccated on 20 October 2016, and soybean was sown between the terraces over the palisade grass straw under no-tillage. The soybean was harvested on 9 March 2017, and sunn hemp was then sown as a cover crop.

#### 4.2.5. 2017–18 Season

The sunn hemp was desiccated on 7 November 2017, and maize was sown between the terraces on the sunn hemp straw on 24 November 2017, under no-tillage. Palisade grass was sown on 14 December 2017; two rows were sown between rows of the maize crop.

Table S4 in the Supplementary Files summarizes the crop history in the system, and Table S5 in the Supplementary Files provides the details of the nutrients applied.

### 4.3. Sampling and Analysis

In October 2022, six years after thinning of the eucalyptus, soil samples were taken for chemical analysis and determination of fertility [45]. The samples were collected from trenches that were opened with the aid of a backhoe at three random points in each plot; each trench had a depth of 2.0 m and a width of 8.0 m. From each trench, three subsamples were collected at 0, 2.0, 4.0, and 6.0 m from the eucalyptus tree rows from the central portion of each of the following layers: 0–0.05, 0.05–0.1, 0.1–0.2, 0.2–0.4, 0.4–0.6, 0.6–0.8, and 0.8–1.0 m. The nine subsamples from each layer of each plot were homogenized and pooled to form a composite sample of the layer of the plot.

Soil pH was evaluated in 0.01 M CaCl_2_·2H_2_O solution, and total acidity (H^+^ + Al^3+^) was determined by titration using 1 N calcium acetate (C_4_H_6_CaO_4_) solution, pH 7.0. S-SO_4_^2−^ content was determined by the calcium phosphate (Ca_3_(PO_4_)_2_) method, and the levels of P, K^+^, Ca^2+^, and Mg^2+^ in the soil were determined by extraction with ion-exchange resin [45]. These results were used to calculate the base saturation (BS) based on the relationship between the content of exchangeable bases in the soil (Ca^2+^, Mg^2+^, and K^+^) and the cation exchange capacity (CEC) in cmol_c_ dm^−3^.

## 5. Conclusions

This study evaluated the effects of three percentages of the thinning of the eucalyptus hybrid Urograndis H-13 on soil chemical fertility in an agrosilvopastoral system. Six years after eucalyptus thinning, the changes in the soil chemical attributes were greater in the 100% eucalyptus thinning treatment than in the 0% and 50% eucalyptus thinning treatments. These changes included increases in the soil P, K^+^, Ca^2+^, and Mg^2+^ contents, soil pH, and BS and a decrease in the total acidity and Al^3+^ content throughout the soil profile (0–1.0 m), especially near the eucalyptus line (0 and 2 m).

## Figures and Tables

**Table 1:** Selected chemical and physical characteristics of the soil before the initiation of the experiment.

Depth	P	OM ^(1)^		pH	K^+^		Ca^2+^		Mg^2+^		H^+^ + Al^3+ (2)^		BS ^(3)^
m	mg dm^−3^	g dm^−3^			----------------- cmol_c_ dm^−3^ ------------------								%
0–0.2	7	17		5.2	0.03		0.18		0.08		0.16		64
0.2–0.4	3	15		5	0.02		0.16		0.06		0.16		59
	Sand					Silt				Clay			
	----------------------------------g kg^−1^------------------------------------												
0–0.2	815					104				81			
0.2–0.4	783					142				75			
	M ^(4)^		µ ^(5)^			TP ^(6)^		BD ^(7)^		>2 mm ^(8)^		MWD ^(9)^	
	-------------- m^3^ m^−3^ ---------------							kg dm^−3^		%		mm	
0–0.2	0.03		0.34			0.38		1.59		57.88		2.76	
0.2–0.4	0.03		0.34			0.37		1.58		52.26		2.61	

^(1)^ organic matter; ^(2)^ total acidity pH 7.0 (H^+^ + Al^3+^); ^(3)^ base saturation = 100(Ca^2+^ + Mg^2+^ + K^+^/CEC pH 7.0); ^(4)^ macroporosity; ^(5)^ microporosity; ^(6)^ total porosity; ^(7)^ bulk density; ^(8)^ percentage of aggregates > 2 mm; ^(9)^ mean weight diameter.

## Data Availability

Data are contained within the article and Supplementary Materials.

## Supplementary Materials

The following supporting information can be downloaded at: https://www.mdpi.com/article/10.3390/plants13213050/s1, Table S1. Values of the F test for changes in soil chemical attributes at depths of 0–0.05, 0.05–0.1, and 0.1–0.2 m. Table S2. Values of the F test for changes in soil chemical attributes at depths of 0.2–0.4, 0.4–0.6, and 0.6–0.8 m. Table S3. Values of the F test for changes in soil chemical attributes at a depth of 0.8–1.0 m. Table S4. Crop history from September 2009 to March 2023. Table S5. Nutrient amounts used between September 2009 and March 2023.
