# Supplementary material for: Changes in Soil Chemical Attributes in an Agrosilvopastoral System Six Years After Thinning of Eucalyptus
Source: Plants (Basel). 2024 Oct 31;13(21):3050. doi: 10.3390/plants13213050 (PMC11548533; doi:10.3390/plants13213050)
Supplement: Supplementary file 1 [file plants-13-03050-s001.zip › plants-3184932-supplementary.pdf]

Table S1. Values of the F test for changes in soil chemical attributes at depths of 0–0.05, 0.05–0.1, and 0.1–0.2 m.

Source of variation	P	S	OM[6]	pH	K^+	Ca^{2+}	Mg^{2+}	$H^+ + Al^{3+}$ [7]	Al	BS[8]
					0–0.05 m					
Factor1-F1[1]	18.8855**	21.5103**	5.6321**	17.5834**	18.5029**	2.6846^{ns}	2.1771^{ns}	11.8069**	61.5786**	9.782**
Factor2-F2[2]	14.083**	6.9344**	17.7796**	40.5745**	9.792**	16.8514**	15.094**	21.3136**	89.9579**	35.0209**
Interaction F1xF2	4.6792**	5.1411**	5.9122**	2.1893^{ns}	7.7577**	3.3201*	6.5881**	0.6129^{ns}	48.6492**	3.6499**
LSD1[3]	6.77	3.89	9.53		0.84	11.69	6.20		0.57	9.07
LSD2[4]	7.46	4.29	10.51		0.92	12.89	6.83		0.63	10.00
CV[5]%	24.22	29.30	20.89	3.16	19.16	28.82	22.62	13.42	28.73	7.97
					0.05–0.1 m					
Factor1-F1	4.9777*	0.489^{ns}	0.1561^{ns}	4.9777*	0.7332^{ns}	4.6446*	2.6997^{ns}	0.3435^{ns}	72.9771**	0.5038^{ns}
Factor2-F2	47.5735**	7.3076**	5.3129**	47.5735**	33.5408**	61.201**	10.4754**	45.5072**	472.0813**	12.9522**
Interaction F1xF2	7.2438**	8.7449**	7.2103**	7.2438**	9.0939**	3.9033**	3.0299*	6.8731**	24.8189**	1.3116^{ns}
LSD1	0.37	2.90	5.53	0.37	0.52	3.91	4.90	4.65	0.69	
LSD2	0.40	3.20	6.10	0.40	0.57	4.31	5.41	5.13	0.76	
CV%	4.23	22.12	18.57	4.23	16.01	14.87	29.88	13.28	22.56	16.50
					0.1–0.2 m					
Factor1-F1	1.4177^{ns}	10.8757**	4.5839*	5.7209**	20.0441**	18.0097**	0.4604^{ns}	4.0348*	27.9248**	6.0781**
Factor2-F2	7.3502**	6.2716**	5.5957**	18.8655**	10.5676**	12.9257**	9.6874**	20.7241**	172.785**	18.9433**
Interaction F1xF2	8.1443**	9.3766**	2.8742*	3.071*	4.355**	3.4025*	11.5326**	2.4681*	65.7704**	3.9107**
DMS1	1.82	1.96	2.19	0.51	0.36	4.05	2.42	6.17	0.95	13.81
DMS2	2.00	2.16	2.41	0.56	0.39	4.46	2.67	6.81	1.05	15.23
CV%	22.40	16.09	10.18	5.95	14.11	20.61	17.53	18.62	20.95	15.25

[1] Percentage of thinning. [2] Sampling positions in relation to the eucalyptus line. [3] Least significant difference (LSD) of percentage of thinning within sampling positions. [4] LSD of sampling positions within the thinning percentages. [5] Coefficient of variation. [6] Organic matter. [7] Total acidity pH 7.0 ($H^+ + Al^{3+}$). [8] Base saturation (BS) = 100($Ca^{2+} + Mg^{2+} + K^+$/CEC pH 7.0). * Significant at the 0.05 probability level. ** Significant at the 0.01 probability level.

Table S2. Values of the F test for changes in soil chemical attributes at depths of 0.2–0.4, 0.4–0.6, and 0.6–0.8 m.

Source of variation	P	S	OM[6]	pH	K^+	Ca^{2+}	Mg^{2+}	$H^+ + Al^{3+}$ [7]	Al	BS[8]
0.2–0.4 m										
Factor1-F1[1]	1.3252^{ns}	5.4235**	0.3942^{ns}	26.0577**	0.1503^{ns}	36.7047**	7.7196**	12.7095**	12.959**	21.7046**
Factor2-F2[2]	5.6948**	1.8226^{ns}	2.0181^{ns}	9.9458**	5.0839**	6.7952**	2.4892^{ns}	17.7763**	8.3422**	14.6878**
Interaction F1xF2	4.177**	7.8172**	1.6871^{ns}	1.465^{ns}	4.0668**	2.8205*	1.5861^{ns}	3.1184*	2.759*	1.5209^{ns}
LSD1[3]	1.45	3.10			0.65	3.57		5.17	2.53	
LSD2[4]	1.60	3.42			0.72	3.94		5.70	2.79	
CV[5]%	27.83	25.03	10.72	5.07	23.93	19.00	24.37	16.83	66.67	13.78
0.4–0.6 m										
Factor1-F1	10.114**	32.1144**	5.4033**	44.2594**	1.5209^{ns}	31.5543**	29.0928**	5.0701*	143.69**	34.8038**
Factor2-F2	3.4737*	16.2942**	1.5852^{ns}	45.3835**	16.1811**	8.9692**	4.7199**	56.3817**	123.8592**	38.5934**
Interaction F1xF2	12.8158**	16.7968**	1.0343^{ns}	5.7411**	19.8936**	2.7434*	4.8963**	2.3313^{ns}	28.2694**	1.1998^{ns}
LSD1	0.89	2.41		0.28	0.39	0.39	2.44		0.68	
LSD2	0.98	2.66		0.31	0.43	0.43	2.69		0.75	
CV%	19.99	16.31	8.66	3.28	14.43	16.87	16.82	10.04	18.68	7.91
0.6–0.8 m										
Factor1-F1	1.1864^{ns}	25.6032**	5.8228**	0.1068^{ns}	7.768**	0.1902^{ns}	1.7403^{ns}	0.9769^{ns}	7.1827**	0.8281^{ns}
Factor2-F2	3.0472*	9.4919**	7.1353**	40.4254**	7.1175**	1.4658^{ns}	1.9343^{ns}	25.1987**	97.8888**	14.491**
Interaction F1xF2	6.1292**	10.0949**	4.4479**	6.4028**	2.1249^{ns}	2.1015^{ns}	1.0666^{ns}	8.9634**	17.7733**	4.5971**
DMS1	0.89	7.48	1.47	0.37				3.51	1.14	10.27
DMS2	0.98	8.25	1.62	0.41				3.87	1.26	11.32
CV%	29.81	25.70	9.32	4.25	28.88	19.98	30.28	13.06	31.89	10.66

[1] Percentage of thinning. [2] Sampling positions in relation to the eucalyptus line. [3] Least significant difference (LSD) of percentage of thinning within sampling positions. [4] LSD of sampling positions within the thinning percentages. [5] Coefficient of variation. [6] Organic matter. [7] Total acidity pH 7.0 ($H^+ + Al^{3+}$). [8] Base saturation (BS) = 100($Ca^{2+} + Mg^{2+} + K^+$/CEC pH 7.0). * Significant at the 0.05 probability level. ** Significant at the 0.01 probability level.

Table S3. Values of the F test for changes in soil chemical attributes at a depth of 0.8–1.0 m.

Source of variation	P	S	OM(6)	pH	K^+	Ca^{2+}	Mg^{2+}	$H^+ + Al^{3+}$ (7)	Al	BS(8)
	0.8–1.0 m									
Factor1-F1(1)	9.8594**	25.6032**	3.9589*	10.6423**	25.7916**	8.8416**	0.9192^{ns}	3.0427^{ns}	9.3213**	14.3538**
Factor2-F2(2)	2.75^{ns}	9.4919**	2.3413^{ns}	45.3682**	18.5094**	3.0389*	0.4906^{ns}	6.0958**	109.1312**	13.107**
Interaction F1xF2	2.9844*	10.0949**	2.79*	2.8331*	4.1048**	1.1342^{ns}	0.3995^{ns}	1.4518^{ns}	6.0228**	2.8964*
LSD1(3)	0.67	7.48	1.42	0.28	0.49				0.79	5.39
LSD2(4)	0.74	8.25	1.56	0.31	0.54				0.87	5.94
CV(5)%	29.48	25.70	9.74	3.14	20.00	18.45	26.06	12.67	41.73	5.51

(1) Percentage of thinning. (2) Sampling positions in relation to the eucalyptus line. (3) Least significant difference (LSD) of percentage of thinning within sampling positions. (4) LSD of sampling positions within the thinning percentages. (5) Coefficient of variation. (6) Organic matter. (7) Total acidity pH 7.0 ($H^+ + Al^{3+}$). (8) Base saturation (BS) = 100($Ca^{2+} + Mg^{2+} + K^+$/CEC pH 7.0). * Significant at the 0.05 probability level. ** Significant at the 0.01 probability level.

Table S4. Crop history from September 2009 to March 2023.

Growing Season	September–March	April–August
2009–10	Millet/soybean	Sunn hemp
2010–11	Maize + palisade grass	Maize + palisade grass
2011–16	Palisade grass	Palisade grass
2016–17	Soybean	Sunn hemp
2017–18	Maize + Palisade grass	Palisade grass
2018–23	Palisade grass	Palisade grass

Table S5. Nutrient amounts used between September 2009 and March 2023.

Growing Season	N (kg ha^{-1})	P (kg ha^{-1})	K (kg ha^{-1})
2009–10	15.0	124.0	60.0
2010–11	116.4	91.0	86.4
2011–12	45.0	0	0
2012–13	33.0	0	0
2013–14	100.0	0	0
2014–15	50.0	0	0
2015–16	25.0	0	0
2016–17	16.0	80.0	80.0
2017–18	129.2	88.2	104.4
2018–23	0	0	0
